# Creatinine Fluctuations Forecast Cross-Harvest Kidney Function Decline Among Sugarcane Workers in Guatemala

**DOI:** 10.1016/j.ekir.2020.06.032

**Published:** 2020-07-12

**Authors:** Miranda Dally, Jaime Butler-Dawson, Richard J. Johnson, Lyndsay Krisher, Diana Jaramillo, Kira L. Newman, Lee S. Newman

**Affiliations:** 1 Center for Health, Work, & Environment, Colorado School of Public Health, University of Colorado Anschutz Medical Campus, Aurora, Colorado, USA; 2 Department of Environmental and Occupational Health, Colorado School of Public Health, University of Colorado Anschutz Medical Campus, Aurora, Colorado, USA; 3 Division of Renal Diseases and Hypertension, School of Medicine, University of Colorado Anschutz Medical Campus, Aurora, Colorado, USA; 4 Internal Medicine Residency Program, University of Washington, Seattle, Washington, USA; 5 Department of Epidemiology, Colorado School of Public Health, University of Colorado Anschutz Medical Campus, Aurora, Colorado, USA; 6 Division of Pulmonary Sciences and Critical Care Medicine, School of Medicine, University of Colorado Anschutz Medical Campus, Aurora, Colorado, USA

**Keywords:** agricultural workers, AKI, chronic kidney disease of unknown origin, forecasting, kidney function decline, occupational health

## Abstract

**Background:**

Chronic kidney disease of unknown origin (CKDu) is an epidemic that disproportionately affects young agriculture workers in hot regions. It has been hypothesized that repeated acute kidney injury (AKI) may play a role in the development of disease.

**Methods:**

Latent class mixed models were used to identify groups of Guatemalan sugarcane harvesters based on their daily changes in creatinine over 6 consecutive days in 2018. Exponential smoothing state space models were used to forecast end-of-season creatinine between the identified groups. Percent change in estimated glomerular filtration rate (eGFR) across the harvest was compared between groups.

**Results:**

Twenty-nine percent (n = 30) of the 103 workers experienced repeated severe fluctuations in creatinine across shift. The model with multiplicative error, multiplicative trend, and multiplicative seasonality was able to accurately forecast end-of-season creatinine in the severe group (mean percentage error [MPE]: −4.7%). eGFR of workers in the severe group on average decreased 20% across season compared to 11% decline for those in the moderate group (95% confidence interval for difference: −17% to 0%).

**Conclusions:**

Daily fluctuations in creatinine can be used to forecast end-of-season creatinine in sugarcane harvesters. Workers who experience repeat severe daily fluctuations in creatinine, on average, experience a greater reduction in kidney function across the season.

## Graphical abstract

**Figure:**
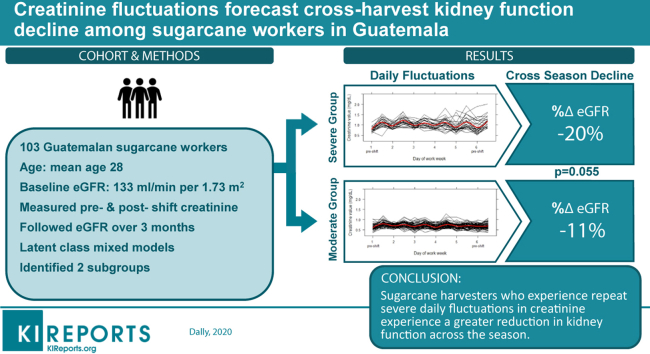

CKDu is an epidemic that disproportionately affects young agricultural workers in hot regions around the world.^1^ Unlike other forms of chronic kidney disease (CKD), CKDu has not been linked to the traditional risk factors, such as diabetes and hypertension. A recent systematic review in Central America found that to date the only personal risk factors with strong evidence of association with disease are male sex, family history of CKD, amount of water consumed while at work, and residing and working at low altitude.^2^

One of the leading hypotheses is that CKDu occurs when people perform heavy work in hot climates while under a state of dehydration,^1^^,^^3^, ^4^, ^5^ leading to kidney injury experienced across the work shift and across the harvest season. It has been shown that agricultural workers experience both acute cross-shift changes in kidney function^5^, ^6^, ^7^, ^8^ as well as cross-harvest changes.^9^, ^10^, ^11^ Researchers have speculated that recurrent AKI results in CKDu, although evidence is sparse.^12^, ^13^, ^14^, ^15^ We hypothesize that acute changes in kidney function can be used to forecast who will develop CKDu, in which case early detection and primary prevention strategies can be deployed.

In this article we used statistical modeling techniques to identify patterns of daily kidney function changes, as measured by serum creatinine, over the course of 6 days, in a cohort of sugarcane harvesters in Guatemala. We then assessed the accuracy of these patterns in forecasting the end-of-season creatinine of workers. To determine the impact of daily creatinine fluctuations on longer term changes in kidney function across the season, we compared the percent change in eGFR 3 months later between the identified groups.

## Methods

### Study Population

We conducted a study among male sugarcane harvesters who were employed at a large agribusiness in Guatemala during the 2017–2018 sugarcane harvest. Individuals in this study were recruited from 2 randomly selected work groups and provided informed consent. Ethics review and approval for the study was received from the Colorado Multiple Institutional Review Board (COMIRB #17-1328) and ZUGUEME Comité Ética Independiente in Guatemala.

Cutting sugarcane is considered very heavy work involving swinging a machete to cut the stalk a few centimeters above ground level, followed by lifting, trimming, and stacking the cane. Sugarcane harvesters in our population cut on average 6 tons of sugarcane per day with average temperatures during the work shift upward of 30 °C.^16^ Workers are typically in the field for 10-hour work shifts (7:00 to 17:00) with three 20-minute rest breaks and a 1-hour lunch break. Workers in our study were provided access to clean water; electrolytes; rest periods; shade; personal protective equipment, such as shin guards, sun hats, and goggles; and education on topics including hydration, rest, hygiene, safe sexual practices, nutrition, and risks of using drugs and nonprescription medicines.^5^^,^^7^^,^^11^

### Study Design

Although the 2017–2018 harvest started in November 2017, primary data collection occurred at mid-harvest, during a 2-week period in January 2018. January was selected as the study month to reduce loss to follow-up, as most workers who leave the workforce do so before January. ^16^ It is standard practice for workers to work 6 consecutive days followed by a rest day. Starting on day 1 of each group’s work week, each worker was followed for a consecutive 6-day period allowing us to capture information on the entirety of their work week (study days 1–6). For each day in the study, pre- and post-shift measurements of kidney function were collected.

**Figure 1:**
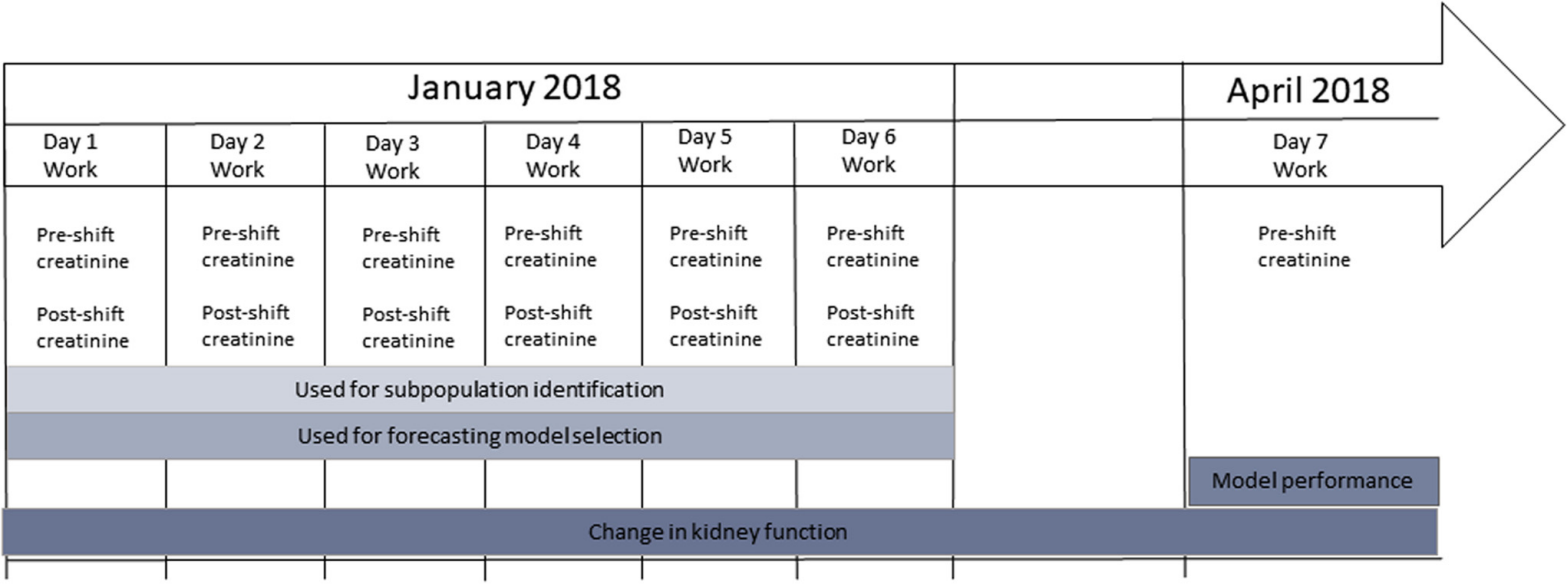
Summary of data collection and analysis design.

Follow-up data collection occurred in April 2018, near the end of the harvest, and included pre-shift measurements of kidney function (study day 7). Data collections occurred in the fields in which the workers were working. Study design is summarized in Figure 1.

### Kidney Function

Serum creatinine is a measurement of kidney function and is used to determine whether individuals experience AKI. AKI is defined as an increase in a venous sample of serum creatinine by ≥0.3 mg/dl or to ≥ 1.5 times baseline.^17^ Either definition was used to indicate AKI in our study.^7^ To calculate AKI, we measured capillary creatinine levels at the start of the work shift and the end of the work shift using the point-of-care Nova Statscan (Nova Biomedical Corporation, Waltham, MA). Based on previous comparisons between venous and capillary samples of post-shift point-of-care creatinine measures, we applied an adjustment factor of 0.7775 to all the post-shift capillary point-of-care creatinine values to relate them to venous values.^18^ Preliminary data have shown that there is good agreement between venous and capillary samples of pre-shift measurements requiring an adjustment factor of 1, or no adjustment, to relate capillary creatinine values to venous values taken before the start of work.^19^

One of the main criticisms of defining AKI across the work shift is that the observed fluctuations in creatinine may be due to already having reduced kidney function or other factors that might cause a slight acute increase in creatinine, such as muscle injury,^20^ rather than an indicator of true kidney injury measured by reduction in eGFR. To assess the association of repetitive cross-shift AKI with longer term kidney function decline, we calculated the percent decline in eGFR for individuals between January and April. To calculate eGFR we used the Chronic Kidney Disease Epidemiology Collaboration formula.^21^ Race was considered “non-Black” and all workers were male.

### Statistical Analysis

Previous research on CKDu, as well as other forms of CKD, suggest that subpopulations may be at higher risk for accelerated decline in kidney function.^22^ Therefore, a 3-step approach was used to analyze the data. First, we identified latent subpopulations based on daily changes in creatinine from pre-shift to post-shift over the 6-day study. We then identified and used the patterns in creatinine fluctuation experienced by these subpopulations to forecast end-of-season creatinine measured in April. To assess changes in eGFR from January to April, we compared the average percent change in eGFR between identified subpopulations. All statistical analyses were done using R version 3.4.3.^23^

#### Step 1: Subpopulations

To identify patterns of cross-shift creatinine changes, we used latent class mixed models to model the shape of pre-shift to post-shift to pre-shift changes in kidney function over the course of the 6-day work week.^24^ Time was treated as time of day (1 = day 1 pre-shift creatinine and 1.5 = day 1 post-shift creatinine measurement). Longitudinal change in creatinine was modeled with a cubic time trend with random term for time at the individual level. Unconditional models were used to determine class-membership. Splines with 5 equidistant nodes were used as the link function. To assess classification error, we examined the posterior classification probabilities that summarize the mean of the posterior probability among individuals classified into each subpopulation along with the proportion of individuals classified into each subpopulation with posterior probabilities above 0.7, 0.8, and 0.9.^25^ Full specifications for our model selection are provided in the Supplementary Methods. The package “lcmm”^25^ was used for this analysis.

#### Step 2: Forecasting

Each individual was assigned a probability for subpopulation assignment from the latent class mixed model. Individuals were assigned to the subpopulation with which they had the highest probability. Data were then aggregated within each subpopulation by taking the average creatinine value within the subpopulation at each time point, totaling 13 time points (12 pre- and post-shift values on days 1–6 and 1 pre-shift value on day 7).

For each of the subpopulations, we fit exponential smoothing state space models^26^^,^^27^ using data from study days 1 to 6. To account for the pre-shift to post-shift pattern in the data, we defined the seasonality as pre-shift to post-shift and post-shift to pre-shift by treating the frequency of the time series as 2. The exponential smoothing state space models were specified using 3 parameters: error type, trend type, season type. Error types were either additive (A) or multiplicative (M). Trend and season types were A, M, or none (N). Defining the error, trend, or season as additive would indicate that the effect appears to increase with the mean whereas to define as multiplicative would indicate that the size of the effect is directly proportional to the mean.^28^ Having no trend or seasonality would indicate a flat line.

To assess the appropriate forecast model for each of the subpopulations, we fit the 12 allowed combinatorial models for each subpopulation for the pattern of day 1 through day 6. We calculated the accuracy of each model by comparing the forecasted April creatinine with the observed April creatinine. We used the MPE as our fit statistics to compare models. The MPE is calculated as the average percentage from which the forecasted values differ from the actual values. In the case of end-of-season creatinine, a negative MPE would indicate that the forecasted value, on average, overestimated the observed value. The farther away from 0 would indicate a greater over- or underestimate depending on if the MPE was negative or positive, respectively. We chose to select the model with the negative MPE closest to 0% as the best-performing model, as this model would error on the side of overestimating cross-season change. We errored on the side of overestimating end-of-season creatinine because from a surveillance and intervention perspective we would rather predict an overestimate of the severity of kidney function decline rather than underestimate it. Should all the MPE values be positive, we select the model with the lowest MPE. The package “forecast”^29^ was used for this analysis.

#### Step 3: eGFR Change

To determine differences in percent change in eGFR from January to April between the subpopulations, we calculated the observed individual percent change in eGFR for each worker. The average percent change in eGFR was then averaged among workers in each subpopulation and compared using Student’s *t*-test. This step was done independent of the results from Step 2.

## Results

There were 107 male workers who consented to participate in the study. Of these, 4 workers were present only during 1 day of the 6-day period and were excluded from the analysis. The average age of the study population was 28 years (SD: 7). The average day 1 pre-shift creatinine was 0.66 mg/dl (SD: 0.15) and the average April creatinine was 0.88 mg/dl (SD: 0.22). All workers had normal eGFR (eGFR > 90 ml/min per 1.73 m^2^) at pre-shift on day 1 of the study. The average day 1 pre-shift eGFR was 132.8 ml/min per 1.73 m^2^ (SD: 15.6) and the average April eGFR was 115.9 ml/min per 1.73 m^2^ (SD: 20.2), with the average January to April change in eGFR of −14% (SD: 16%).

### Subpopulations

**Figure 2:**
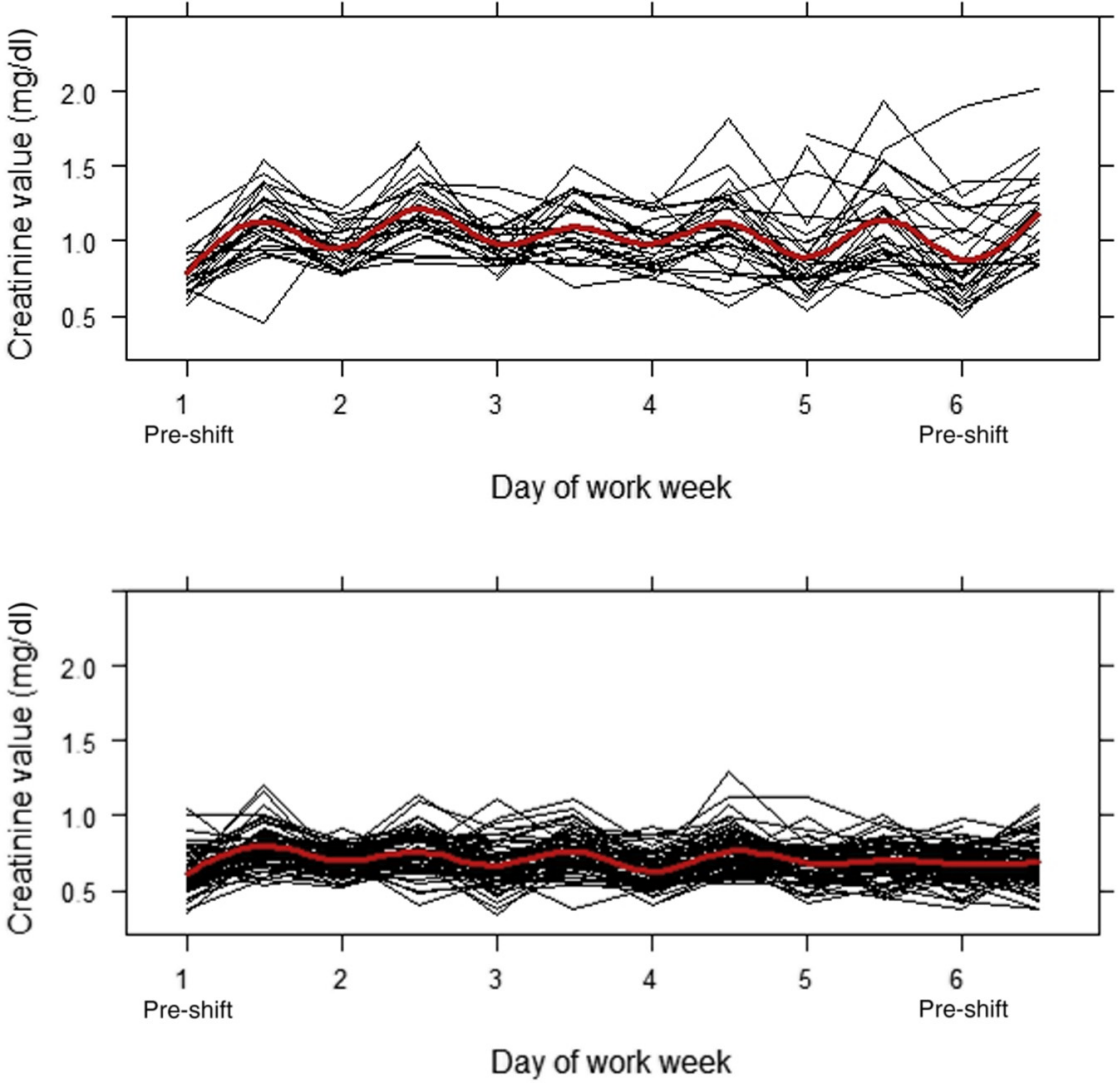
Individual pre- and post-shift serum creatinine measurements for 103 Guatemalan sugarcane cutters during a 6-day work week in January 2018, stratified by severe (top) and moderate (bottom) daily fluctuation subpopulations. Red line represents a fitted cubic spline to demonstrate the average pattern of change for each subpopulation.

Two unique subpopulations for daily cross-shift changes in creatinine were identified, a severe group and a moderate group. Individuals in the severe group (n = 30; 29%) had higher daily pre-shift measures, greater daily fluctuations between pre- to post-shift creatinine, and higher daily post-shift measures compared with the moderate group (n = 73; 71%) (Figure 2). Posterior classification probabilities were 87% for the severe group and 95% for the moderate group. Of individuals identified in the severe group, 63% had a posterior probability above 90% and 77% had a posterior probability above 70%. For individuals identified in the moderate group, 86% had a posterior probability above 90%, 92% a posterior probability above 80%, and 97% a posterior probability above 70%.

Daily incidence rates of AKI ranged from 23% to 67% in the severe group compared with 2% to 21% in the moderate group (Table 1). Although the moderate group experienced recovery overnight, the severe group did not fully return to pre-shift creatinine levels. The pre-shift creatinine of the severe group worsened over the course of the work week. A summary of the decomposition of the time series of creatinine over the work week for each group is provided in Supplementary Figure 1 (severe group) and Supplementary Figure 2 (moderate group). The average April creatinine was 1.08 mg/dl (SD: 0.27) for the severe group and 0.81 mg/dl (SD: 0.16) for the moderate group (*P*-value for difference: 0.0001).

**Table 1:** Daily pre- and post-shift creatinine measurements, presented as mean (SD), and daily incidence rate of AKI, presented as *n* (%), stratified by subpopulation

	Moderate (*n* = 73; 71%)						Severe (*n* = 30; 29%)					
	Day 1	Day 2	Day 3	Day 4	Day 5	Day 6	Day 1	Day 2	Day 3	Day 4	Day 5	Day 6
Pre-shift creatinine mg/dl	0.62 (0.13)	0.70 (0.09)	0.67 (0.14)	0.63 (0.12)	0.69 (0.12)	0.68 (0.12)	0.78 (0.14)	0.95 (0.14)	0.98 (0.15)	0.98 (0.17)	0.89 (0.30)	0.88 (0.32)
Post-shift creatinine mg/dl	0.80 (0.13)	0.76 (0.14)	0.77 (0.13)	0.76 (0.14)	0.70 (0.13)	0.69 (0.16)	1.14 (0.22)	1.23 (0.21)	1.08 (0.21)	1.13 (0.28)	1.15 (0.30)	1.18 (0.28)
AKI^a^	16 (23)	3 (4)	7 (10)	10 (14)	1 (2)	6 (9)	18 (67)	9 (35)	6 (23)	6 (25)	12 (44)	17 (59)

AKI, acute kidney injury.

a AKI is defined as an increase in serum creatinine by ≥ 0.3 mg/dl or an increase in serum creatinine to ≥1.5 times baseline.

### Forecasting

**Figure 3:**
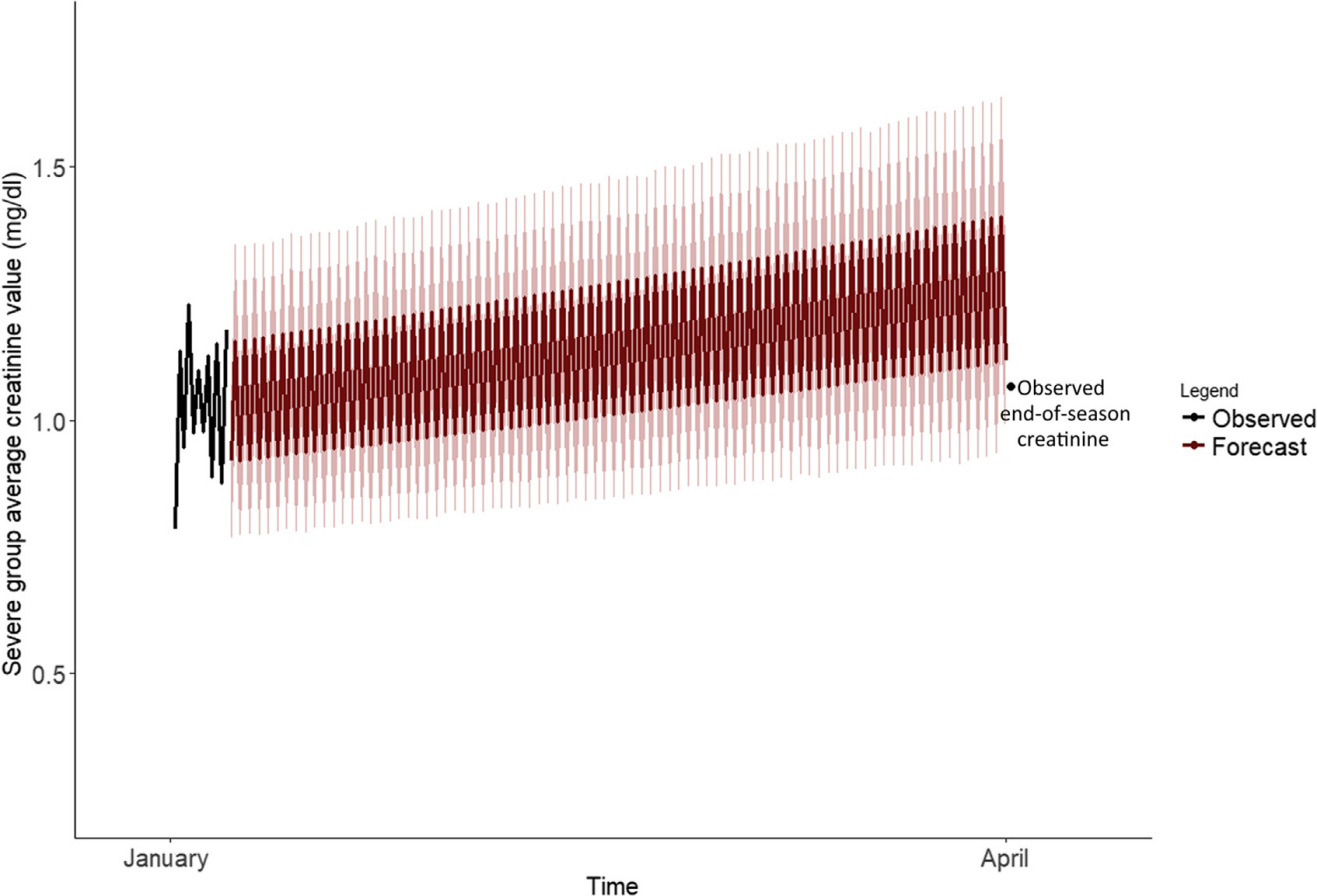
Severe group observed January changes in pre- to post-shift creatine and forecasted cross-harvest change in creatinine based on the MMM exponential smoothing state space model along with 95% confidence bands for projections. Comparison made to the observed April measurement.

The 3 best-performing models for forecasting the April creatinine value of the severe subpopulation were with an additive error and no trend nor seasonality (MPE: 3.4%), with a multiplicative error and no trend or seasonality (MPE: 3.4%), and with a multiplicative error, multiplicative trend, and multiplicative seasonality (MPE: −4.7%). The model with multiplicative error, trend, and seasonality was selected as the final model for the severe group (Figure 3), as this model overestimated end-of-season creatinine, whereas the other 2 models underestimated end-of-season creatinine. A summary of all fit statistics for all tested models for the severe subpopulation is provided in Supplementary Table S1.

**Figure 4:**
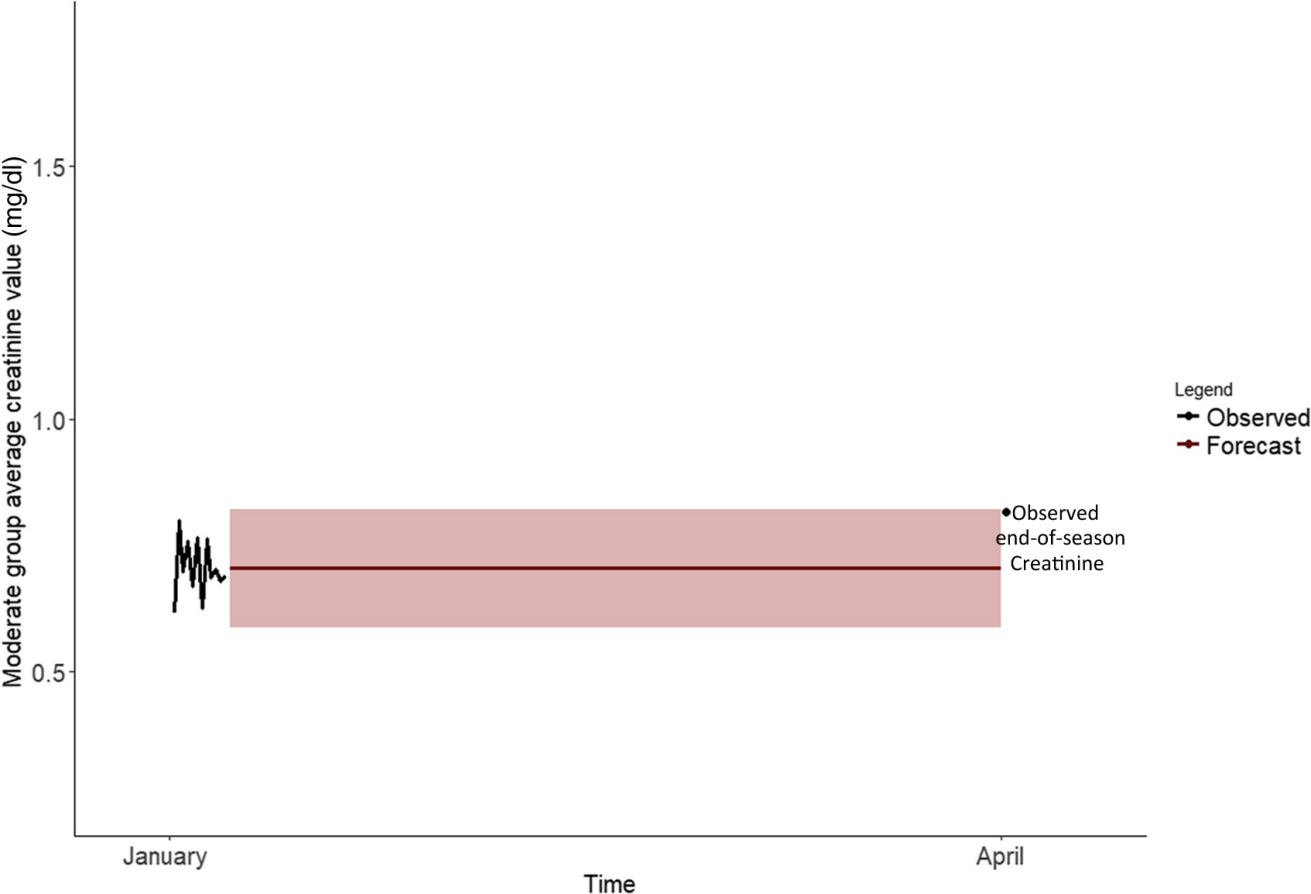
Moderate group observed January changes in pre- to post-shift creatine and forecasted cross-harvest change in creatinine based on the MNN exponential smoothing state space model along with 95% confidence bands for projections. Comparison made to the observed April measurement.

The 3 best-performing models for the moderate subpopulation were with an additive error and no trend or seasonality (MPE: 13.8%), with a multiplicative error and no trend or seasonality (MPE: 13.8%), and with an additive error with no trend and additive seasonality (MPE: 18.7%). The model with a multiplicative error term with no trend or seasonality was selected as the final model for the moderate group to keep the error terms consistent between the subpopulations. This model, which captures no information about trend or seasonality, suggests the best prediction for April in the moderate group is the average of all creatinine values observed over the 6 days (Figure 4). A summary of all fit statistics for all tested models for the moderate subpopulation is provided in Supplementary Table S2.

### Cross-Season eGFR

The average day 1 pre-shift eGFR was 118.8 ml/min per 1.73 m^2^ (SD: 13.9) for the severe group and 137.0 ml/min per 1.73 m^2^ (SD: 13.1) for the moderate group. The average April eGFR was 94.0 ml/min per 1.73 m^2^ (SD: 21.8) for the severe group and 119.8 ml/min per 1.73 m^2^ (SD: 15.5) for the moderate group. The average percent change in eGFR from January to April was a 20% decrease (SD: 19%) for the severe group compared with a 12% decrease (SD: 14%) in the moderate group. On average, the severe group eGFR declined 8% more across the season (95% CI: −17% to 0%) compared with the moderate group (*P*-value: 0.055).

## Discussion

Fluctuations in creatinine between pre- and post-shift over the course of 6 days can be used to forecast end-of-season creatinine in agricultural field workers. Workers who experience repeated severe fluctuations in creatinine across the work shift, on average, experience a greater reduction in eGFR across the season. It has been suggested that due to natural variances in creatinine, cross-shift measurements of kidney function decline are not a reliable way of detecting true reduction of kidney function.^30^ Our data provide evidence that daily increases in creatinine are not occurring at random and suggest that cross-shift changes in creatinine may contribute to observed cross-season declines in kidney function. Interestingly, the 70% of our sample who we identified as only having moderate fluctuations in creatinine also experienced cross-season decline in kidney function, consistent with research in Nicaragua, which found an average decline of 10% in eGFR over the course of 9 weeks among sugarcane harvesters. ^9^ This suggests that there are factors, in addition to daily changes in creatinine, contributing to observed reductions in kidney function.

We observed that the daily pattern in creatinine identified for the severe subpopulation accurately forecasted the observed April creatinine for those individuals. One of the critiques of previous cross-shift studies of AKI is that random variations in serum creatinine may be a significant contributor to AKI diagnosis in the absence of a true reduction of kidney function.^30^ This study addresses this concern by demonstrating that individuals identified in the severe subpopulation had a greater 3-month reduction in eGFR. Although questions remain as to whether this observed decline in eGFR is reversible or leads to CKDu, a recent study in Nicaragua found that of 34 workers who experienced kidney injury, defined as an increase in serum creatinine ≥0.3 mg/dl over baseline to a level ≥1.3 mg/dl across a 6-month harvest, 40% had eGFR declines greater than 30% up to 1 year later.^10^ Given both these findings, more research is needed to determine the cause of severe fluctuations in serum creatinine.

Evidence suggests that AKI and CKD are closely related, with CKD as a risk factor for AKI and AKI as a risk factor for the development of CKD.^31^ However, in the setting of CKDu, the relationship between cross-shift changes in creatinine and the observed declines in kidney function over the course of a harvest season has been questioned.^30^^,^^32^ This is due to physiologic variability of serum creatinine levels after physical activity, where creatinine rises with muscle breakdown and may not reflect a true AKI. In this study, the recurrence of severe fluctuations across the work shift contributed to declines in kidney function measured by eGFR over the course of 3 months. It should be noted that those workers in the severe subpopulation began the harvest with lower eGFR than those in the moderate subpopulation (119 ml/min per 1.73 m^2^ vs. 137 ml/min per 1.73 m^2^, respectively). This finding may indicate that workers in the severe subpopulation are already experiencing early reductions in kidney function, making their kidneys more susceptible to dehydration or nephrotoxic exposures, which could explain the observed increase in cross-shift changes in creatinine and cross-season change in eGFR. This leads us to suggest that cross-shift changes in creatinine may be an early indicator for workers at risk for experiencing more severe cross-harvest change in kidney function.

Interestingly, our forecasting models did not perform well for the moderate group. Because the moderate group saw a marked reduction in incidence of AKI and severity of cross-shift creatinine change as the work week progressed (Supplementary Figure S2), the models did not accurately forecast the observed cross-season decline for this group. It is unclear why we observed a reduction in AKI and less change in creatinine across the shift with this subpopulation. Future research will examine potential occupational and behavioral factors for assignment to the severe versus moderate subpopulations.

### Limitations

First, we were limited in the number of observations that made up our time series. Second, the forecasting in our analysis was dependent on accurate subpopulation classification through the latent class mixed model. Although the latent class mixed model provided good discrimination, evidenced by the high posterior probabilities, there is the potential that workers were misclassified into the severe and moderate groups. Third, the potential for selection bias is present as we recruited workers in January leading to the potential exclusion of workers who may have left the workforce due to health issues.

Although our study shows that repeated severe fluctuations in creatinine are associated with longer term changes in eGFR, we cannot say whether this goes on to cause CKDu, because a probable case of CKDu is defined as 2 abnormal eGFR results (< 60 ml/min per 1.73 m^2^) at least 3 months apart.^33^ Notably, we had only 1 data point with which to assess the validity of the forecast. As shown with the January data, creatinine is variable in this population. Creatinine may also be affected by changes in hydration, diet, or muscle injury.^34^ Although we do not suspect dehydration habits or diet changed between January and April, the reduced creatinine observed in April may be an artifact of the timing of the measurement. In addition, because both muscle mass and GFR determine an individual’s serum creatinine level, using calculations such as the Chronic Kidney Disease Epidemiology Collaboration with only the inclusion of creatinine to estimate GFR may be inaccurate on an individual level.^35^

Although this study suggests that individuals who experience severe fluctuations in creatinine across a work shift are more likely to experience a cross-harvest change in eGFR, future research is required to determine if the observed changes in kidney function across the season are real and sustained and if these losses lead to the development of CKDu.

## Conclusions

Agricultural workers around the world are at risk for declines in kidney function during their daily work shifts and harvest seasons, which may lead to the development of CKDu.^1^ By recognizing that cross-shift changes in creatinine may contribute to these cross-harvest declines in kidney function, we have provided evidence that interventions are needed to reduce cross-shift change in creatinine to both prevent AKI and longer term kidney function decline. Future research is needed to identify risk factors for cross-shift kidney function declines so that workplaces are better positioned to implement programs and interventions to address kidney health and reduce the risk of developing CKDu among their workers.

## Disclosure

All the authors declared no competing interests.
