# Supplementary material for: Creatinine Fluctuations Forecast Cross-Harvest Kidney Function Decline Among Sugarcane Workers in Guatemala
Source: Kidney Int Rep. 2020 Jul 12;5(9):1558–66. doi: 10.1016/j.ekir.2020.06.032 (PMC7486184; doi:10.1016/j.ekir.2020.06.032)

Supplementary Material

Supplementary methods. Latent class mixed model selection

To determine the best functional form for the model, models with linear, quadratic, and cubic terms for time and a random term for time at the individual level were run using linear, beta, and spline link functions. The nine resultant single class models were then compared using the Bayesian Information Criterion (BIC). To determine the number of optimal latent classes, grid searches using 100 random vectors of initial values and a maximum of 15 iterations were used along the range of latent classes 2 through 5 for the best performing functional form. The resulting class models were compared using BIC and the model with the lowest BIC was selected. The R package “lcmm” was used [25]. Posterior classification probabilities ranged from 87% to 95%.

Table S1. Fit statistics for all tested forecasting models in the severe group. Exponential smoothing state space models fit on work days 1 - 6 with fit statistics calculated for pre-shift April. Fit statistics include ME: Mean Error; RMSE: Root Mean Squared Error; MAE: Mean Absolute Error; MPE: Mean Percentage Error; MAPE: Mean Absolute Percentage Error; MASE: Mean Absolute Scaled Error.

	ME	RMSE	MAE	MPE	MAPE	MASE
ANN	0.035949	0.035949	0.035949	3.372347	3.372347	0.155385
MNN	0.03596	0.03596	0.03596	3.373403	3.373403	0.155434
**MMM**	**-0.0501**	**0.050101**	**0.050101**	**-4.69988**	**4.699881**	**0.216553**
MAM	-0.11252	0.112522	0.112522	-10.5556	10.55558	0.486362
MNM	0.156831	0.156831	0.156831	14.71206	14.71206	0.677877
ANA	0.158668	0.158668	0.158668	14.88439	14.88439	0.685818
MNA	0.162974	0.162974	0.162974	15.28836	15.28836	0.704431
MAA	-0.25243	0.252431	0.252431	-23.6802	23.68023	1.091098
AAA	-0.27986	0.279857	0.279857	-26.253	26.25302	1.209643
MAN	-1.05732	1.057318	1.057318	-99.1855	99.18554	4.570104
AAN	-1.47277	1.472767	1.472767	-138.158	138.1583	6.365824
MMN	-1.56135	1.561355	1.561355	-146.469	146.4685	6.748731

Table S2. Fit statistics for all tested forecasting models in the moderate group. Exponential smoothing state space models fit on work days 1 - 6 with fit statistics calculated for pre-shift April. Fit statistics include ME: Mean Error; RMSE: Root Mean Squared Error; MAE: Mean Absolute Error; MPE: Mean Percentage Error; MAPE: Mean Absolute Percentage Error; MASE: Mean Absolute Scaled Error.

	ME	RMSE	MAE	MPE	MAPE	MASE
ANN	0.11291	0.11291	0.11291	13.81834	13.81834	1.319836
**MNN**	**0.112911**	**0.112911**	**0.112911**	**13.81848**	**13.81848**	**1.31985**
ANA	0.152895	0.152895	0.152895	18.71191	18.71191	1.787238
MNA	0.155513	0.155513	0.155513	19.03225	19.03225	1.817835
MNM	0.155927	0.155927	0.155927	19.08288	19.08288	1.82267
AAN	0.200758	0.200758	0.200758	24.56949	24.56949	2.346715
MAN	0.227176	0.227176	0.227176	27.80262	27.80262	2.655522

MMN	0.480848	0.480848	0.480848	58.84799	58.84799	5.620771
MAA	0.484445	0.484445	0.484445	59.28821	59.28821	5.662818
MMM	0.521306	0.521306	0.521306	63.79947	63.79947	6.093704
MAM	0.719843	0.719843	0.719843	88.09717	88.09717	8.41446
AAA	0.746603	0.746603	0.746603	91.37206	91.37206	8.727256

Figure S1. Decomposition of the 6-day work week creatinine time series for the severe group. Presented as data, fitted seasonality, trend, and remainder.

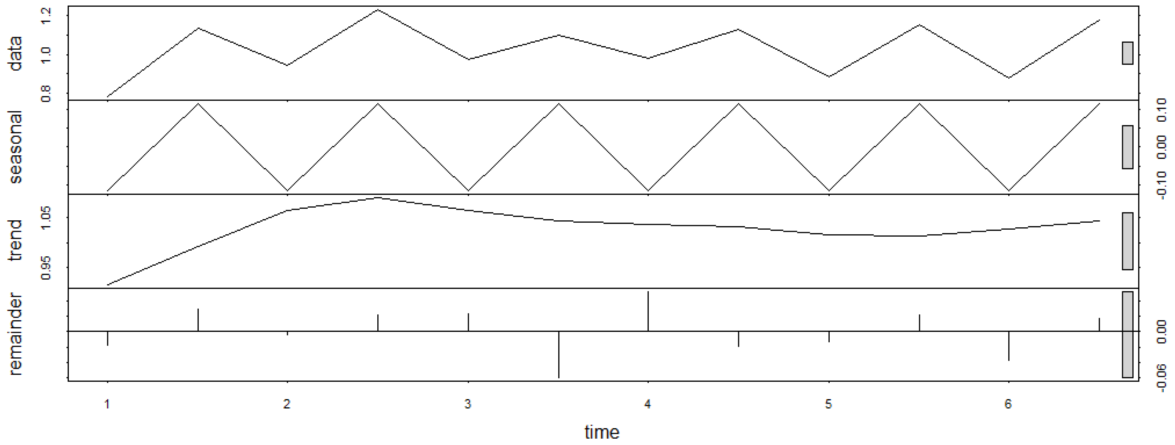

Figure S2. Decomposition of the 6-day work week creatinine time series for the moderate group. Presented as data, fitted seasonality, trend, and remainder.

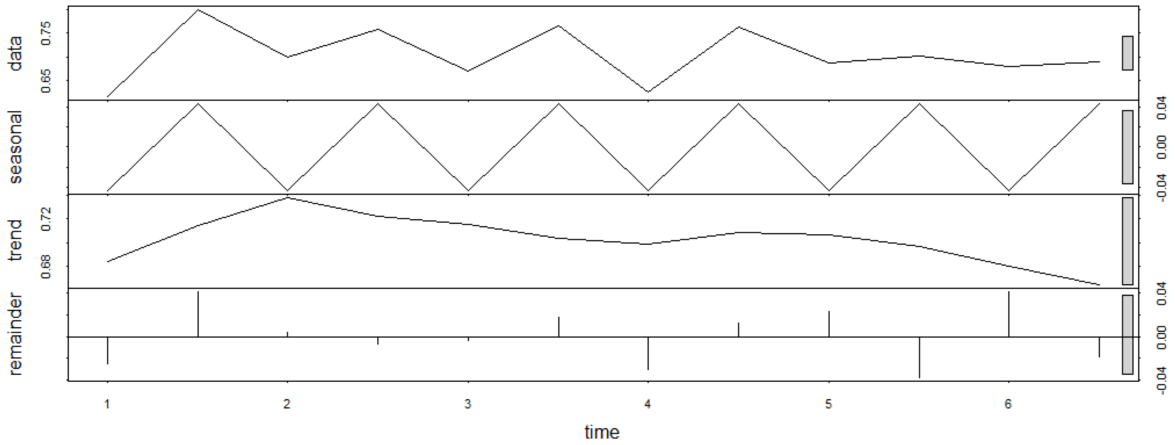

Supplement: Supplementary File (PDF) [file mmc1.pdf]
